# Education, survival and avoidable deaths in cancer patients in Finland

**DOI:** 10.1038/sj.bjc.6605861

**Published:** 2010-08-17

**Authors:** A Pokhrel, P Martikainen, E Pukkala, M Rautalahti, K Seppä, T Hakulinen

**Affiliations:** 1 Finnish Cancer Registry, Institute for Statistical and Epidemiological Cancer Research, Pieni Roobertinkatu 9, FI-00130, Helsinki, Finland; 2 Department of Sociology, FI-00014 University of Helsinki, Helsinki, Finland; 3 Cancer Society of Finland, Pieni Roobertinkatu 9, FI-00130, Helsinki, Finland

**Keywords:** survival after cancer, social inequality, educational position, equity, avoidable deaths

## Abstract

**Background:**

Relative survival after cancer in Finland is at the highest level observed in Europe and has, in general, been on a steady increase. The aim of this study is to assess whether the high survival is equally shared by different population subgroups and to estimate the possible gains that might be achieved if equity prevailed.

**Materials and method:**

The educational level and occupation before the cancer diagnosis of patients diagnosed in Finland in 1971–2005 was derived from an antecedent population census. The cancers were divided into 27 site categories. Cancer (cause)-specific 5-year survival proportions were calculated for three patient categories based on the educational level and for an occupational group of potentially health-conscious patients (physicians, nurses, teachers etc.). Proportions of avoidable deaths were derived by assuming that the patients from the two lower education categories would have the same mortality owing to cancer, as those from the highest educational category. Estimates were also made by additionally assuming that even the mortalities owing to other causes of death were all equal to those in the highest category.

**Results:**

For almost all the sites considered, survival was consistently highest for patients with the highest education and lowest for those with only basic education. The potentially health-conscious patients had an even higher survival. The differences were, in part, attributable to less favourable distributions of tumour stages in the lower education categories. In 1996–2005, 4–7% of the deaths in Finnish cancer patients could have potentially been avoided during the first 5-year period after diagnosis, if all the patients had the same cancer mortality as the patients with the highest educational background. The proportion would have also been much higher, 8–11%, if, in addition, the mortality from other causes had been the same as that in the highest educational category.

**Interpretation:**

Even in a potentially equitable society with high health care standards, marked inequalities persist in cancer survival. Earlier cancer diagnosis and the ability to cope within the health care system may be a partly relevant explanation, but personal habits and lifestyles also have a role, particularly for the cancer patients’ mortality from other causes of death than cancer.

Finland has one of the highest relative survival for cancer patients in Europe (Capocaccia *et al*, 2009). Studies in Denmark and the UK, countries with lower relative survival than in Finland, showed fairly large and consistent survival differences between educational and social groups (Shack *et al*, 2007; Dalton *et al*, 2008; Rachet *et al*, 2008). Increased survival does not necessarily mean the eradication of survival differences between subgroups (Coleman *et al*, 2008). Studies in Finland have demonstrated systematic differences by social class in cancer survival (Karjalainen and Pukkala, 1990; Auvinen, 1992; Auvinen *et al*, 1995; Dickman *et al*, 1997). It is important to determine whether these differences exist and their size, to plan actions on the basis of their likely causes from which they might have emerged. The causes for educational survival differences may be related to, e.g., behavioural characteristics, tumour stage at diagnosis or the health care system (Dalton *et al*, 2008).

The aim of the present study is to explore systematic social differences in cancer survival in Finland and how it has changed over time. Educational level was selected as the main variable defining the social subgroups, as it has been shown to strongly predict cancer survival in Denmark (Dalton *et al*, 2008) and as its definition has remained comparable over time. The impact of these differences was demonstrated by numbers and proportions of potentially avoidable deaths under the assumption of cause-specific survival in all educational groups being equal to that in the subgroup with the highest education.

## Materials and methods

Patients diagnosed with cancer in 1971–2005 and registered by the countrywide Finnish Cancer Registry were linked, through the use of the unique personal identity codes with the population censuses done every 5 years in 1970–2000, to obtain information on patients’ educational levels and occupations (Pukkala *et al*, 2009). Persons born in 1906–80 were included in the analyses. Using the most recent antecedent information from census, the educational levels were divided into three categories according to the highest attained educational degree or certificate as follows: basic (lasting typically <10 years), secondary (10–12 years) and high education (13 years or more). Patients below 25 years of age were excluded as their educational level was not necessarily the final one.

Occupational details were used to create a supplementary group that should be particularly conscious of their health and cancer care possibilities, namely persons in medical and nursing work, teaching work, research and physical education. In comparative analyses involving in this category, such individuals were excluded from the educational categories (mainly from the high educational category) to form a fourth group. Cancers were divided into 27 site categories (Table 1). The patients were followed up from diagnosis until death, emigration or to the end of 2005, whichever came first: for a few (0.13%), the follow-up was terminated owing to emigration.

The main outcome measure of the patients was the cancer (cause)-specific 5-year survival, where deaths owing to causes other than the cancer of the patients are counted as events censoring the follow-up time. The Cancer Registry corrects, when necessary, the official cause-of-death information, as it has more information than the person signing the death certificate (Hakulinen and Teppo, 1977). The Registry receives, on average, five notifications per cancer case, and registration is regarded as reliable both in diagnostic accuracy and completeness (Teppo *et al*, 1994; Curado *et al*, 2007).

The relative survival ratio, another measure preferred by population based cancer registries in estimating the cause-specific survival without relying on the quality of death certificates, was used for comparison (Ederer *et al*, 1961). Here, mortality owing to competing causes of death other than the patient's cancer is estimated as an overall mortality from the general population life tables by sex, age and calendar period. A linkage between a 20% random sample of the individuals from the censuses of 1970–2000 with the register of deaths and emigrations made it possible to also obtain general population mortality rates in the three educational categories. Thus, differences in general mortality by educational category were taken into account in relative survival analyses. For comparison, relative survival analyses without accounting for educational level were also conducted. The estimation of relative survival was based on the method of Hakulinen (1982).

Traditional direct age-standardisation was used for survival comparisons between the educational groups, as the groups differed in age structures (Pokhrel and Hakulinen, 2008); age structure of all patients diagnosed in 1971–2005 was used as a standard for each site. The ages used in the standardisation were 25–44, 45–54, 55–64, 65–74 and 75 years and more. This method maintained the interpretability of the standardised figures and the comparability between calendar periods, genders and educational categories within a cancer site but not between sites. Results were calculated for patient diagnosed in the periods 1971–85, 1986–95 and 1996–2005.

To study and control simultaneously for all prognostic variables, the regression model of Hakulinen and Tenkanen (1987) was used for the cancer (cause)-specific and relative survival analyses. For the former, the expected survival was defined to be uniformly one. The models included five annual follow-up intervals, sex, age (the same categories as in age-standardisation), calendar period (the three periods mentioned) and educational level (three categories). In some analyses, the tumour stage was also included as a covariate. Localised tumours were always classified separately and those remaining were divided into 1–4 categories (regional metastases; distant metastases; non-localised, extent unknown; unknown) depending on the site; the unknown stage category comprised of 10–20% of tumours.

Interactions between age and follow-up year, and age and stage were applied in all models including age and stage to allow for non-proportional cause-specific or excess hazards by these variables (Dickman *et al*, 2004). Cause-specific survival model results are reported as relative risks (RRs) of cause-specific death for a given category compared with a chosen reference category. The corresponding quantities for relative survival models, the relative risks of (cancer-related) excess mortality or the relative excess risk (RER) of death were also derived.

Numbers and proportions of potentially avoidable deaths were calculated for each educational group by assuming that the age- and sex-specific hazards of dying were equal to those in the high educational category by cancer site and cause of death (cancer of the patients, other causes of death). First, the assumption was made for cancer-specific mortality only. Second, the mortality owing to the competing risks of death was, in addition, assumed to be on the level observed in the high educational category. The numbers of avoidable deaths for all sites combined were obtained by summing the site-specific numbers. The theory of competing risks of death (Chiang, 1968) was used to obtain the disease-specific (‘crude’) probabilities of death needed in a hypothetical situation of reduced cause-specific mortality.

## Results

The educational level could be identified from the censuses for 99.2% of patients; those with unknown education were excluded. Altogether, 526 143 patients were included in the analyses (Table 1). Patients with basic education tended to be older than those with a high education, and the educational level increased in the population by calendar time.

**Figure 1:**
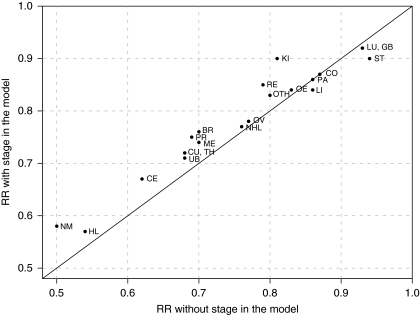
Risk ratios for cancer death of patients diagnosed in 1971–2005 (high *vs* basic education) without and with accounting for stage, by site (sites: OE=oesophagus; ST=stomach; CO=colon; RE=rectum; LI=liver; GB=gallbladder; PA=pancreas; LU=lung; ME=skin, melanoma; NM=skin, non-melanoma; BR=breast; CE=cervix uteri; CU=corpus uteri; OV=ovary; PR=prostate; KI=kidney; UB=urinary bladder; TH=thyroid; HL=Hodgkin's lymphoma; NHL=non-Hodgkin's lymphoma; OTH=other cancers as specified in Table 1). The model accounts for gender where applicable. The diagonal indicates equality of the two RRs. Results are shown for sites with model convergence only.

**Figure 2:**
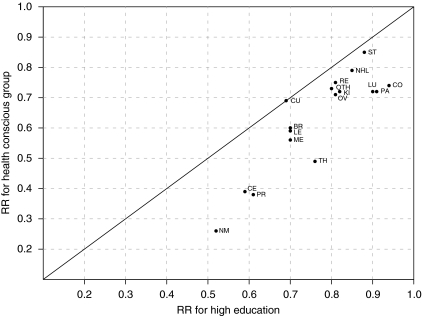
Risk ratios for cancer death high *vs* basic education and health-conscious occupational group *vs* basic education and for patients diagnosed in 1996–2005, by site (sites: ST=stomach; CO=colon; RE=rectum; PA=pancreas; LU=lung; ME=skin, melanoma; NM=skin, non-melanoma; BR=breast; CE=cervix uteri; CU=corpus uteri; OV=ovary; PR=prostate; KI=kidney; TH=thyroid; NHL=non-Hodgkin's lymphoma; LE=leukaemia; OTH=other cancers as specified in Table 1). The model accounts for gender where applicable. Persons in the health-conscious occupational group have been removed from the education categories. The diagonal indicates equality of the two RRs.

The group with a high educational level had a higher age-standardised cancer-specific 5-year survival than that with basic educational level, almost without exception (Table 1). The age-specific and, thereby, also the age-standardised 5-year cancer-specific survival proportions and their differences could not be estimated for every educational group in 1996–2005 for five sites in males and four sites in females. Statistical modelling showed that the higher survival among the highly educated had persisted over calendar time. Differences in RRs of cancer-specific mortality between the educational categories were larger when the stage was not included in the model, and existed only when that RR was under 0.85 (Figure 1). In other words, when there was a larger mortality difference related to educational level, it was partly accounted for by differences in stage distribution, almost regardless of the site. The attenuation of the relative differences varied strongly but was, with the exception of testicular and kidney cancers, quite modest. The health-conscious occupational group still clearly had a more favourable cancer-specific survival than the rest of the high education category (Figure 2). The RRs of this group were also somewhat attenuated when the stage was adjusted for by the model compared with the results derived without adjusting for the stage.

The relative survival analysis, where the baseline general mortality had been calculated specific to the educational category, mostly gave similar results (RER) to those (RR) of the cause-specific analyses (results not shown). Not using comparable baseline mortality with respect to educational category resulted in larger differences between the RERs and the RRs, which also held true when stage was taken into account in the model.

If in the most recent period 1996–2005, the cancer-specific mortality of all male and female patients equalled that of the high education group, 8% of cancer deaths in patients diagnosed at ages 25–64 years during the first 5 years after diagnosis are theoretically avoidable (Table 2). For ages 65–89, the corresponding proportion is higher, 10% these proportions are lower, 7%, when prostate and breast cancers are excluded. When the same estimation is done for the first 10 years after diagnosis, the numbers and proportions of avoidable cancer deaths decrease. At 25–64 years, the proportions of avoidable deaths were fairly stable over time. In contrast, in the first 5-year follow-up period after diagnosis, there has been a clear increase in them at ages 65–89 years, even when prostate and breast cancers are excluded. There has been an increasing trend in avoidable cancer deaths for 10-year follow-up period after diagnosis, which almost disappears after excluding prostate and breast cancers. The numbers and proportions of potentially avoidable deaths varied greatly by cancer site (Table 2). Proportions exceeding 10% were obtained for cancers of the skin, breast, female genital organs, prostate and urinary organs, whereas practically no or few avoidable deaths were obtained for stomach and lung cancers.

The avoidable cancer deaths will contribute to an increase in the number of persons exposed to competing causes of death, so, there will be negative numbers in the avoidable deaths owing to causes other than the cancer (Table 3, upper panel A). At ages 25–64 years, the total proportion of avoidable deaths is 5–7% in the first 5 years and about 3–5% in the first 10-year period after diagnosis; at ages 65–89 years, the respective proportions are 4–6 and 3%.

By assuming that both the cancer-specific mortality and the mortality owing to other causes would be the same as those observed in patients with high education, the numbers and proportions of avoidable cancer deaths are slightly smaller than those without the assumption of an improved mortality from other causes of death (Table 3, lower panel B). On the other hand, marked numbers of avoidable deaths from other causes are obtained, ranging from 9 to 28%. At ages 25–64 years, avoidable deaths, regardless of the cause, are 8–10% during the first 5 years of follow-up after diagnosis and 7–8% in the first 10 years; at ages 65–89 years, these proportions are 8–11 and 6–8%, respectively.

## Discussion

It has been previously shown and generally accepted that cancer incidence is strongly influenced by a person's social position or education, as the risk factors of cancer have not evenly distributed (Pukkala, 1995; Dalton *et al*, 2008). It is less easy to accept that cancer survival should depend on these factors as this may indicate differential access to care. Nordic countries, among them Finland, have been emphasising equality among residents as far as health care services are concerned. Cancer control plans have been made in Finland since 1952 to secure equal rights and potential access to services (Finnish Cancer Committee, 1984). Our study suggests that this goal has not been reached.

Social group differences in cancer survival have been seen wherever it has been studied (Kogevinas *et al*, 1997; Mackillop *et al*, 1997; Rosso *et al*, 1997; Singh *et al*, 2003; Madison *et al*, 2004; Dejardin *et al*, 2006; Shack *et al*, 2007; Dalton *et al*, 2008; Rachet *et al*, 2008). Several factors related to risk profiles, tumour characteristics or the health system might lie behind these survival differences.

Risk-factor related risks of death may well be working to different extents for patient survival in different groups (Dalton *et al*, 2008). It is quite conceivable that cancer survival, not only survival with respect to competing causes of death, will depend on factors like smoking, alcohol use and nutritional status, which may also influence the available treatment options.

In a country with good equity in health services, it may sound implausible that health services are better targeted towards the more educated, but the less educated may well be less aware of early symptoms and may experience a delay in diagnosis (Auvinen *et al*, 1995; Neal and Allgar, 2005; Hansen *et al*, 2008). Indeed, when the stage distribution was adjusted for, the cancer-specific survival figures of the educational groups were closer.

Early detection by the prostate-specific antigen (PSA) test will increase the apparent survival for prostate cancer (Schröder *et al*, 2009), and higher educated persons may have more intense PSA diagnostics than others. There is also an increase in survival for breast cancer patients related to mammographic screening (Berry *et al*, 2005), and that may be associated with the education of patient due to differences in the proportion of participation. A higher education may help in navigating within the health system thus enabling better and more timely care. For example, a higher education of the patient can improve the patient–doctor interaction and the ability to follow care regimens. The fact that the health-conscious patient group (e.g., doctors, nurses, teachers) clearly had the highest cancer-specific survival figures points out that much is achievable, provided that their cancer outcomes could be achieved for everyone. Although higher educated patients may have better financial resources to obtain additional care from the private sector, in Finland this did not exist during the period studied.

The numbers and proportions of avoidable deaths have been used as a measure of what possibly could be achieved if cancer patients’ relative survival could be raised to an optimal level defined by a higher survival in other subgroups (Dickman *et al*, 1997) or other populations (Abdel-Rahman *et al*, 2009). These quantities should be estimated regardless of the cause, as some of the persons saved from cancer death are likely to die from competing causes of death even within a 5- or 10-year period after diagnosis. The savings do not last long, as the competing-risk mortality will wear them off rapidly, depending on the age of the patients. Rather than ‘avoidable’ the term could be ‘postponable’ deaths, and person-years saved (Hakulinen and Teppo, 1976) or expected life years lost per patient might be more useful (Hakama and Hakulinen, 1977; Seppä and Hakulinen, 2009).

## Conclusion

Clear educational differences in cancer patient survival are still observed in Finland, despite the level of survival being internationally very high and regardless of the very strong improvement in survival over the past decades.

## Figures and Tables

**Table 1:** Age-standardised 5-year cancer-specific survival proportions for the cancer patients diagnosed in Finland in 1996–2005 by site, sex and educational level (high, secondary, basic) and differences (in % units) between the high and basic categories, by site and period. Patients' site-specific age distributions in 1971–2005 have been used as site-specific standards (both genders combined)

		**Males**				**Females**				
**Site**	**ICD** **-10 code**	**High**	**Sec**	**Basic**	**Diff**	**High**	**Sec**	**Basic**	**Diff**	**Number (1971–2005)**
Lip	C00	98.5	98.3	97.0	1.5	96.4	99.2	97.9	−1.5	4212
Oesophagus	C15	19.9	NA	9.5	10.4	NA	NA	NA	NA	5687
Stomach	C16	28.8	25.2	24.0	4.7	29.2	30.0	29.9	−0.7	26 958
Colon	C18	54.8	57.9	54.5	0.3	60.1	61.1	55.8	4.3	28 759
Rectum	C19–20	59.8	55.7	52.7	7.1	62.4	57.1	56.8	5.6	19 448
Liver	C22	NA	NA	NA	NA	NA	NA	7.5	NA	6445
Gallbladder	C23–24	NA	15.9	NA	NA	9.4	8.8	NA	NA	6693
Pancreas	C25	NA	NA	NA	NA	NA	3.6	2.6	NA	19 328
Nose	C30–31	NA	NA	NA	NA	62.8	64.2	57.9	4.8	960
Lung	C33–34	10.6	9.5	9.2	1.3	18.8	13.5	12.3	6.5	66 014
Skin, melanoma	C43	82.7	78.2	77.4	5.3	91.9	88.6	84.5	7.4	14 750
Skin, non-melanoma	C44	98.0	94.6	95.8	2.1	97.6	98.8	95.5	2.1	15 383
Soft tissue	C48–49	67.4	NA	47.1	20.3	62.6	60.2	60.7	1.9	3468
Breast	C50	—	—	—	—	89.4	87.1	84.9	4.5	78 292
Cervix uteri	C53	—	—	—	—	78.5	69.0	63.3	15.1	5563
Corpus uteri	C54	—	—	—	—	87.8	83.7	82.2	5.6	17 270
Ovary	C56	—	—	—	—	53.2	47.0	44.5	8.6	12 948
Prostate	C61	87.4	84.1	80.3	7.0	—	—	—	—	59 308
Testis	C62	93.4	95.8	91.4	2.0	—	—	—	—	1727
Kidney	C64–65	61.1	58.9	55.2	5.9	62.1	63.6	60.8	1.3	18 117
Urinary bladder	C67–68	80.7	78.2	76.8	3.9	83.3	77.8	69.7	13.5	18 037
Thyroid	C73	84.2	85.9	82.6	1.5	93.9	92.6	91.1	2.8	8066
Hodgkin's lymphoma	C81	86.8	NA	83.4	3.3	86.2	86.3	82.5	3.7	3099
Non-Hodgkin's lymphoma	C82–85, C96	59.8	52.4	51.1	8.7	65.8	59.4	56.6	9.2	18 818
Multiple myeloma	C90	40.5	36.3	25.4	15.1	37.4	29.5	29.3	8.1	7023
Leukaemia	C91–95	49.3	47.9	40.1	9.1	45.2	38.8	39.1	6.0	11 865
Other cancers		39.2	40.0	33.3	5.8	44.5	39.6	34.7	9.8	47 905
Total										526 143

Abbreviation: NA=not estimable; sec=secondary; ICD=international classification of diseases; Diff=differences. Total numbers of cancer patients in 1971–2005 by site are also given.

**Table 2:** Number (*N*) and proportion (%) of potentially avoidable cancer deaths in Finland by period, age and site in a hypothetical situation where all patients would have the same cancer-specific mortality as those with a high education

	**1971–1985**				**1986–1995**				**1996–2005**			
	**5-year**		**10-year**		**5-year**		**10-year**		**5-year**		**10-year**	
**Age/site**	***N***	**%**	***N***	**%**	***N***	**%**	***N***	**%**	***N***	**%**	***N***	**%**
*25–64 years*												
Stomach	64	1	87	2	−5	0	19	1	−8	−1	−30	−2
Colon and rectum	162	5	197	6	324	15	339	14	83	3	35	1
Lung	332	3	320	3	24	0	26	0	239	4	214	4
Skin, melanoma	189	27	228	26	61	12	118	19	143	31	224	36
Skin, non-melanoma	36	59	43	63	21	76	21	61	10	29	15	38
Breast	358	10	534	11	530	22	516	14	356	17	387	11
Female genital organs	537	20	512	17	208	13	241	13	197	13	216	11
Prostate	96	11	75	7	141	16	136	12	303	27	369	20
Urinary organs	390	20	428	19	183	13	220	14	37	3	−58	−4
Leukaemia	62	4	99	6	6	1	46	5	150	23	144	18
Non-Hodgkin's lymphoma	102	8	39	3	107	10	115	8	195	18	104	7
Other sites	778	7	618	5	544	7	508	6	481	6	405	4
Total	3105	7	3180	7	2143	8	2305	8	2188	8	2026	7
Total excluding. prostate and breast	2652	7	2571	6	1472	6	1653	6	1528	7	1270	5
												
*65–89 years*												
Stomach	121	3	48	1	131	3	112	2	192	5	186	5
Colon and rectum	168	6	243	8	495	9	463	8	504	8	395	6
Lung	69	1	66	1	201	2	192	2	180	1	109	1
Skin, melanoma	36	17	20	8	63	16	72	15	102	17	39	6
Skin, non-melanoma	−3	−4	3	5	46	33	60	37	93	43	102	45
Breast	224	18	253	15	550	28	718	27	616	26	408	12
Female genital organs	222	15	214	13	311	12	293	11	599	22	506	17
Prostate	137	8	39	2	541	15	617	13	1397	31	1764	25
Urinary organs	243	16	241	14	438	16	467	15	490	15	487	13
Leukaemia	27	3	67	6	−24	−2	−20	−1	23	1	97	5
Non-Hodgkin's lymphoma	141	17	176	19	193	9	171	8	466	17	351	12
Other sites	290	3	357	4	785	5	744	5	1351	7	1293	6
Total	1675	5	1727	5	3730	7	3889	7	6014	10	5737	9
Total excluding prostate and breast	1314	5	1435	5	2638	6	2554	5	4001	7	3565	6

**Table 3:** Number (*N*) and proportion (%) of potentially avoidable deaths due to cancer and other causes in cancer patients in Finland in 1996–2005, by age, in two hypothetical situations: (A) all patients would have the same cancer-specific mortality and (B) same cancer and competing-risk mortality as those with a high education

	**Cancer**				**Other**				**Total**			
	**5-year**		**10-year**		**5-year**		**10-year**		**5-year**		**10-year**	
**Age at cancer diagnosis**	***N***	**%**	***N***	**%**	***N***	**%**	***N***	**%**	***N***	**%**	***N***	**%**
*A. Same cancer-specific mortality*												
*25–64 years*												
Total	2188	8	2026	7	−167	−5	−330	−5	2021	7	1696	5
Total excluding prostate and breast	1528	7	1270	5	−148	−6	−266	−6	1380	5	1004	3
*65–89 years*												
Total	6014	10	5737	9	−1371	−6	−2480	−7	4642	6	3257	3
Total excluding prostate and breast	4001	7	3565	6	−1062	−7	−1575	−7	2939	4	1990	3
												
*B. Same cancer-specific and competing-risk mortality*												
*25–64 years*												
Total	1984	8	1703	6	963	27	1365	22	2947	10	3068	8
Total excluding prostate and breast	1339	6	986	4	713	28	957	23	2052	8	1943	7
*65–89 years*												
Total	5035	8	4093	6	4054	17	3867	10	9089	11	7960	8
Total excluding prostate and breast	3191	6	2478	4	2186	15	2054	9	5377	8	4532	6
